# Association between long-range temporal correlations in intrinsic EEG activity and subjective sense of identity

**DOI:** 10.1038/s41598-020-79444-2

**Published:** 2021-01-11

**Authors:** Kazumi Sugimura, Yasuhiro Iwasa, Ryota Kobayashi, Tatsuru Honda, Junya Hashimoto, Shiho Kashihara, Jianhong Zhu, Kazuki Yamamoto, Tsuyoshi Kawahara, Mayo Anno, Risa Nakagawa, Kai Hatano, Takashi Nakao

**Affiliations:** 1 Graduate School of Humanities and Social Sciences, Hiroshima University, 1-1-1, Kagamiyama, Higashi-Hiroshima, Hiroshima, 739-8524 Japan; 2 Graduate School of Education, Hiroshima University, Hiroshima, Japan; 3 Faculty of Education, Hiroshima University, Hiroshima, Japan; 4 Faculty of Liberal Arts and Science, Osaka Prefecture University, Osaka, Japan

**Keywords:** Personality, Psychology

## Abstract

The long-range temporal correlation (LRTC) in resting-state intrinsic brain activity is known to be associated with temporal behavioral patterns, including decision making based on internal criteria such as self-knowledge. However, the association between the neuronal LRTC and the subjective sense of identity remains to be explored; in other words, whether our subjective sense of consistent self across time relates to the temporal consistency of neural activity. The present study examined the relationship between the LRTC of resting-state scalp electroencephalography (EEG) and a subjective sense of identity measured by the Erikson Psychosocial Stage Inventory (EPSI). Consistent with our prediction based on previous studies of neuronal-behavioral relationships, the frontocentral alpha LRTC correlated negatively with identity confusion. Moreover, from the descriptive analyses, centroparietal beta LRTC showed negative correlations with identity confusion, and frontal theta LRTC showed positive relationships with identity synthesis. These results suggest that more temporal consistency (reversely, less random noise) in intrinsic brain activity is associated with less confused and better-synthesized identity. Our data provide further evidence that the LRTC of intrinsic brain activity might serve as a noise suppression mechanism at the psychological level.

## Introduction

In the last two decades, studies have demonstrated that the intrinsic activity of the resting-state brain is not random noise, and instead exhibits scale-free temporal patterns, including slowly decaying autocorrelation, called long-range temporal correlation (LRTC)^1–6^. LRTC has been observed using several neuroimaging modalities, such as electroencephalogram (EEG)^1,7,8^ and functional magnetic resonance imaging (fMRI)^9,10^. LRTC emerges from the neuronal network with balanced excitatory and inhibitory connectivity^11,12^, and is thought to reflect the ability of brain networks to integrate information over extended periods^13–15^.

Such temporal neuronal features link with temporal patterns in behavior^16–18^, including those related to decision making based on one’s internal criteria, known as internally-guided decision making^19,20^. Nakao et al.^20^ reported that participants who showed stronger LRTC in resting-state frontocentral (FCz) alpha (8–13 Hz) EEC showed higher temporal-consistency of the decision in occupational preference judgment tasks (e.g., “Which occupation would you rather? … Lawyer or Designer”). Together with other relevant results from simulation and task-related brain activities, they concluded that participants with stronger frontocentral alpha LRTC (i.e., less random neural dynamics) had less random noise-contaminated internal criteria. Since internal criteria basically correspond to self-knowledge, which is knowledge of one's traits, sense of values, and beliefs^21–26^, this finding is consistent with the idea that our self has a central role in suppressing random noise by integrating information^20,27–29^. In contrast to the relationship between LRTC at the neuronal level and less randomness at the behavioral level^19,20^, the relationship between temporal consistency (reversely, less randomness) in the neuronal and subjective mental levels remains to be elucidated.

A subjective sense of integrated and coherent self across time is conceptualized as identity^30^. This subjective experience of identity consists of two fundamental components: identity synthesis and identity confusion. Identity synthesis is a feeling that one’s self is temporally continuous and consistent and one knows where one is headed (e.g., a sense of knowing their purpose and direction), while identity confusion refers to a feeling of one’s self as temporally changeable and fragmented, and one is indecisive and unable to keep commitments to important life decisions (e.g., a sense of feeling mixed up)^30–33^. Identity synthesis and confusion are not polar opposites but exist simultaneously^31^. Since decision making and identity are assumed to have close relationships^34,35^, a similar relationship between neuronal LRTC and decision making is also expected in the context of identity.

Although the relationship between identity and gray matter volume has been investigated^36^, no previous studies have addressed the relationship between identity and the temporal dynamics of intrinsic brain activity. Besides, neuronal LRTC in both fMRI^37^ and EEG^38^ is known to relate to private self-consciousness, which is the tendency of attending to one’s inner thoughts and feelings^39,40^. However, private self-consciousness does not reflect the aspect of the temporal consistency itself, and has been conceptually distinguished from identity^41–43^.

The present study aims to investigate the link between temporal consistency of intrinsic brain activity (i.e., LRTC) and the subjective sense of temporal consistency of one’s self (i.e., identity). We focused on alpha range LRTC at the frontocentral electrode (FCz) based on previous studies that examined the relationship between neuronal LRTC and internally-guided decision making^19,20^. Detrended fluctuation analysis (DFA)^1,2,44,45^ was applied to quantify LRTC, and the larger DFA exponent (DFAe), within a range of 0.5 to 1.0, was reflective of a high degree of LRTC. The Erikson Psychosocial Stage Inventory (EPSI)^46,47^, which measures the degree of identity synthesis and confusion, was applied to assess identity. We hypothesize that frontocentral alpha LRTC is positively correlated with identity synthesis and/or is negatively correlated with identity confusion. This hypothesis is based on the finding of a previous study^20^ in which participants with stronger frontocentral alpha LRTC (i.e., less random neural dynamics) showed less random noise-contaminated internal criteria (i.e., self-knowledge). Since we had a specific hypothesis, we applied one-tailed statistical tests for those relationships. Nevertheless, we had no specific hypothesis regarding whether identity synthesis or confusion correlates with LRTC, and as such, we examined this point exploratively. That is, we avoided type I error inflation by applying a Bonferroni adjusted *p*-value for the two correlation analyses.

## Results

### LRTC

**Figure 1:**
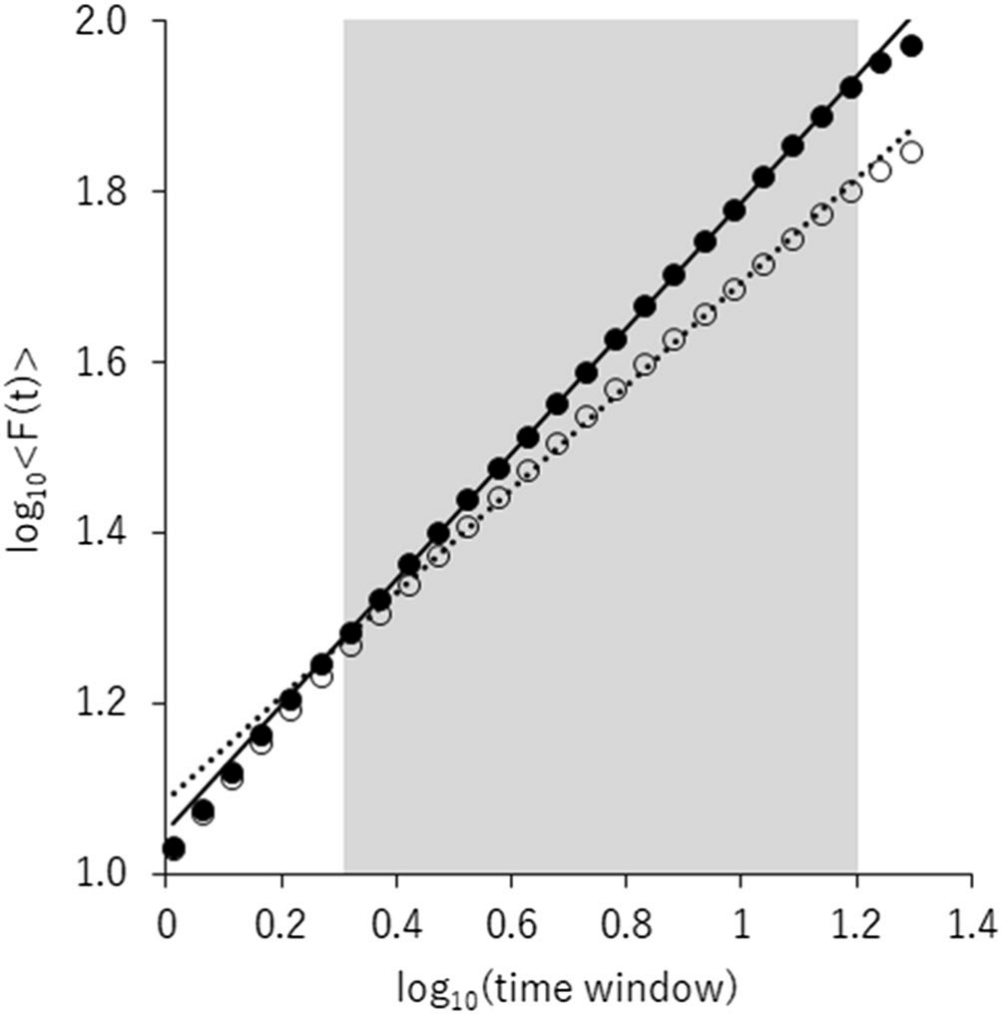
A grand averaged plot of the fluctuation function (F[t]) of alpha range (8–13 Hz) time-series data at FCz of each time window size. The detrended fluctuation analysis exponent (DFAe) corresponds to the slope of the regression line. The filled circles and the solid line present the averaged data across participants and the trend lines using the data within the range of 2–16 s (gray area), respectively. The open circles and dashed line depict the average of all surrogated data and the regression line, respectively.

Since our hypothesis focuses on alpha range LRTC at FCz based on previous studies^19,20^, Fig. 1 shows the results of resting-state alpha range DFAe at FCz. The average DFAe was 0.73 (*SD* = 0.10) at FCz. The ICC was calculated to estimate the reliability of DFAe in the present data, and the ICC of DFAe was found to be acceptable (0.72, 95% CI = 0.55, 0.83).

Surrogated data analysis was conducted in order to confirm whether the DFAe of the present data reflected non-random temporal structure. The grand average of the surrogated alpha DFAe was 0.60 (*SD* = 0.05) at FCz; this result was smaller than the original DFAe values (0.73). The critical value that corresponded to the higher one percentile of the mean surrogated DFAe was 0.61, which was smaller than the observed mean DFAe (0.73). These results indicated that the DFAe of the present data reflected the non-random temporal structure of the phase.

### Identity synthesis and confusion

The mean scores of identity synthesis and confusion were 18.78 (*SD* = 4.89) and 17.82 (*SD* = 4.33), respectively. The rank correlation between identity synthesis and confusion was *rho* = − 0.56 (*p* < 0.001, 95% CI = − 0.70, − 0.37). The Cronbach’s alphas for the present data (*N* = 68) were 0.80 for synthesis and 0.68 for confusion; these values are similar to those reported in previous studies^32,33,47,48^.

In order to confirm the external validity of identity synthesis and confusion, the degree of depressive tendency was measured using the Japanese version^49^ of the Center for Epidemiological Studies Depression Scale (CES-D^50^; Japanese version validated by Shima et al.^51^). The Cronbach’s alpha of the present data was 0.85 for the CES-D. In line with previous studies^52–54^, synthesis and confusion showed negative (*rho* = − 0.51, *p* < 0.001, 95% confidence interval [CI] = − 0.66, − 0.30, and positive (*rho* = 0.54, *p* < 0.001, 95% CI = 0.34, 0.69) correlations with CES-D. Therefore, the external validities of the identity measurements were confirmed.

### Relationship between LRTC and identity

**Figure 2:**
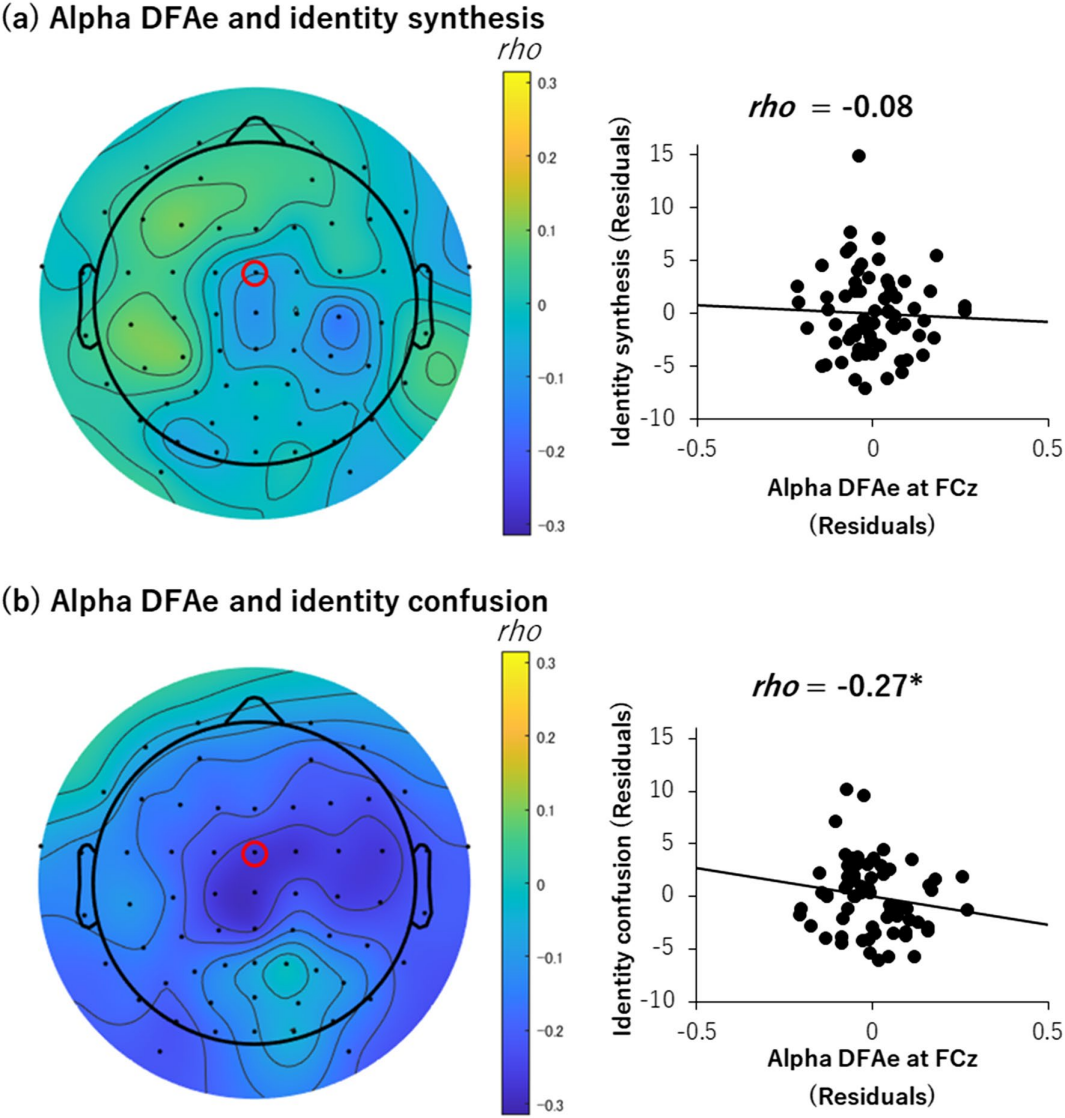
Topo maps and scatterplots of Spearman’s partial correlation analyses. (**a**) Correlation between alpha range DFAe at FCz and identity synthesis. (**b**) Correlation between alpha range DFAe at FCz and identity confusion. *Adjusted *p* < 0.05 (one-tailed). The red circle on each topo map indicates an electrode; the data from which is shown in the scatterplot. Topo maps were created by EEGLAB toolbox (ver.14.1.2b, https://sccn.ucsd.edu/eeglab/index.php)^55^.

Figure 2 shows the correlation between the alpha DFAe at FCz and the identity (synthesis or confusion). Bonferroni correction was applied for the two correlation analyses between DFAe and the two identity factors (synthesis and confusion) in order to avoid type I error inflation. No significant correlation was found between the alpha DFAe and identity synthesis (*rho* = − 0.08, adjusted *p* = 0.53, lower-bound 95% CI = − 0.32, one-tailed) (Fig. 2a); however, identity confusion showed a significant negative correlation (rho = − 0.27, adjusted *p* = 0.03, upper-bound 95% CI = − 0.02, one-tailed) (Fig. 2b) with the DFAe.

The mean surrogated DFAe for each participant showed no significant correlation with identity (synthesis, *rho* = − 0.05, adjusted *p* = 0.73, lower-bound 95% CI = − 0.29, one-tailed; confusion *rho* = − 0.02, adjusted *p* = 0.88, upper-bound 95% CI = 0.23, one-tailed).

**Figure 3:**
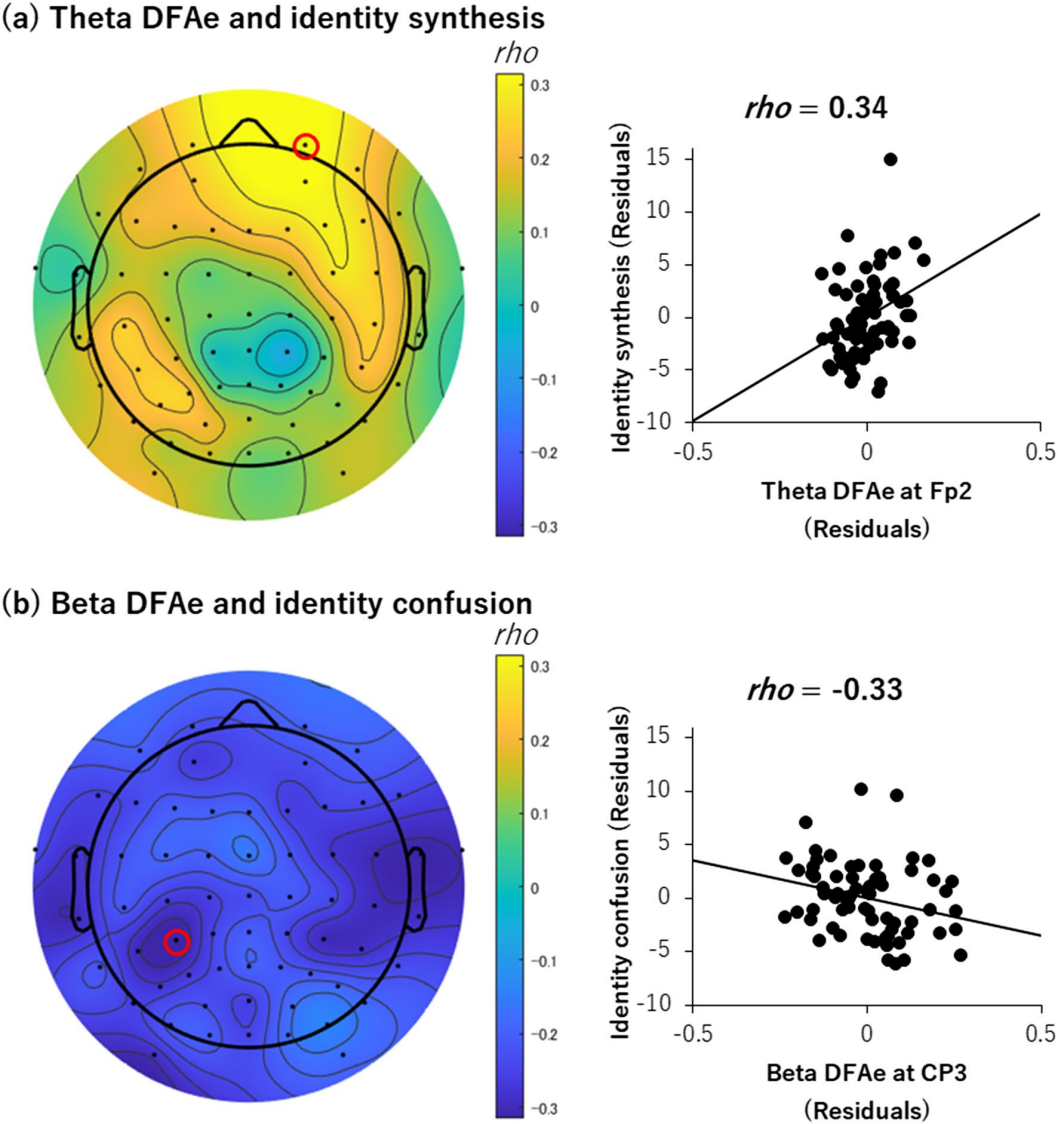
Topo maps and scatterplots of Spearman’s partial correlation analyses for descriptive purposes. (**a**) Correlation between theta range DFAe and identity synthesis. (**b**) Correlation between beta range DFAe and identity confusion. The red circle on each topo map indicates an electrode; the data from which is shown in the scatterplot. Topo maps were created by EEGLAB toolbox (ver.14.1.2b, https://sccn.ucsd.edu/eeglab/index.php)^55^.

For the descriptive purposes, the strongest positive correlation between DFAe and identity synthesis was found in the theta range at Fp2 (*rho* = 0.34, CI = 0.10, 0.54) (Fig. 3a). For identity confusion, the strongest negative correlation with DFAe was found in the beta range at CP3 (*rho* = − 0.33, CI = − 0.54, − 0.09) (Fig. 3b).

All of the significant correlations were retained even when we additionally controlled the degree of depression (CES-D), which was measured to confirm the external validities of identity measurements. Specifically, the significant correlation between alpha LRTC at FCz and identity confusion shown in Fig. 2(b) was retained after controlling for the degree of depression (*rho* = − 0.26, adjusted* p* = 0.046, upper-bound 95% CI = − 0.005, one-tailed). Besides, both the correlations shown in Fig. 3 were retained even when we additionally controlled the degree of depression (for theta LRTC at Fp2 and synthesis, *rho* = 0.32, CI = 0.01, 0.53; for beta LRTC at CP3 and confusion, *rho* = − 0.27, CI = − 0.49, − 0.02).

## Discussion

**Figure 4:**
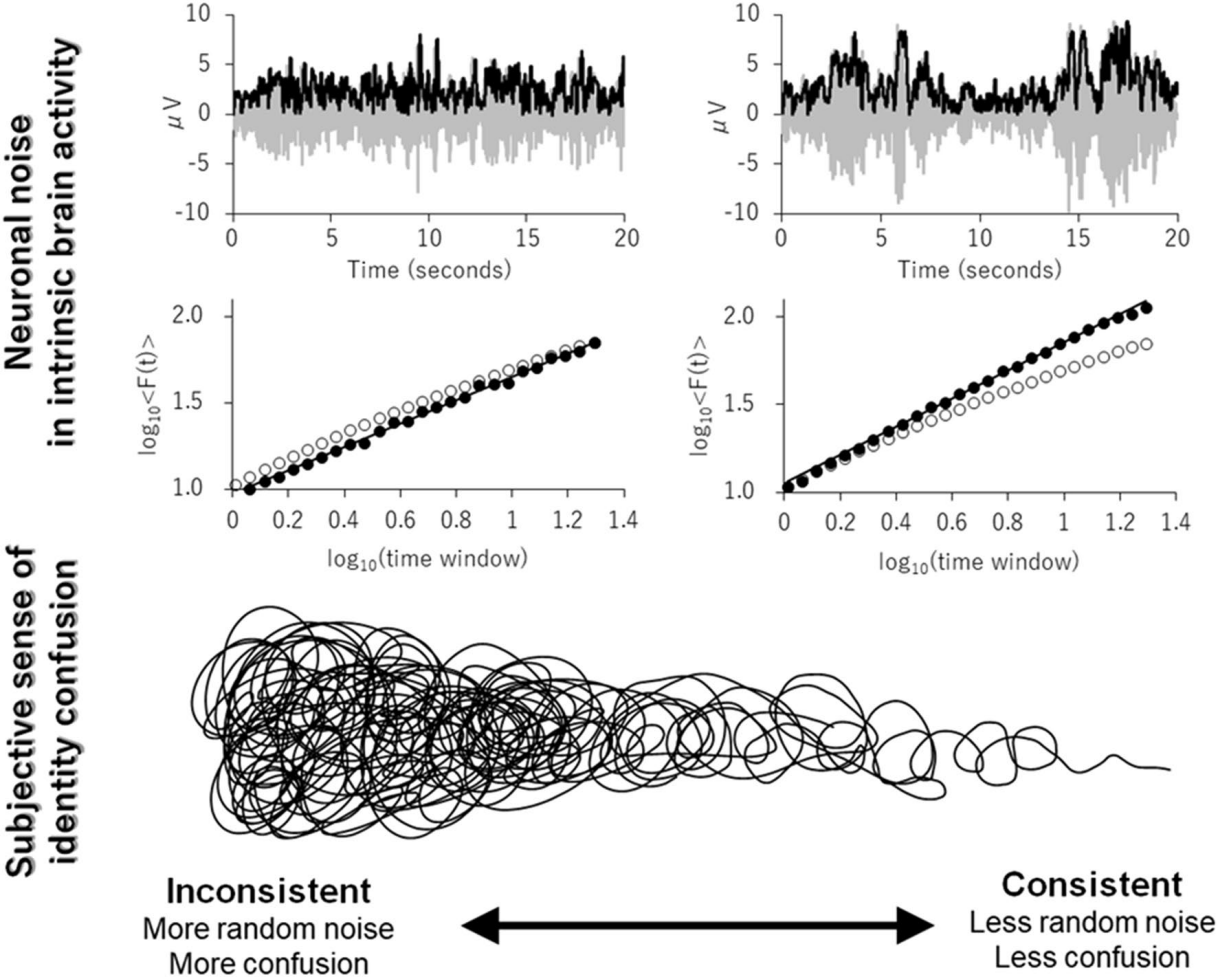
Schematic illustration of the main finding of the present study. The upper row shows examples of the 20 s filtered EEG signal of 8–13 Hz at FCz. The middle row represents the DFAe fluctuation functions. The filled circles represent data, and the open circles, shown as a reference, depict the surrogated data as with Fig. 1. The left panels show data from the participant with DFAe = 0.68 and identity confusion = 25, and the right panels show data from the participant with DFAe = 0.80 and identity confusion = 11. The third row represents a schematic figure of identity confusion.

The present study sought to investigate the relationship between neural temporal consistency and subjective sense of identity. Consistent with our hypothesis, individuals with higher alpha range LRTC in the frontocentral channel had a less subjective sense of identity confusion (Fig. 2(b), see also Fig. 4 for a schematic summary). Although the correlation was not strong (*rho* = − 0.27), this finding is consistent with that of the previous study regarding the relationship between the neuronal and behavioral levels. Specifically, a higher frontocentral alpha LRTC (i.e., less neuronal noise) was associated with high consistency (i.e., less randomness) in occupation preference judgment^20^. Thus, the temporal dynamics reflected in the LRTC of intrinsic brain activity likely have a crucial role in the consistency of self, including the subjective level, namely, a sense of identity.

It is intriguing that the (negative) correlation with frontocentral alpha LRTC was found only in identity confusion, albeit we expected that the (positive) correlation would also be found in identity synthesis. The prediction with respect to the frontocentral alpha was based on the finding of the previous decision making study^20^, in which LRTC was associated with less noise on internal criteria. Given that a result consistent with the prediction was found only in the identity confusion, the small amount of noise contained in the internal criteria may be perceived as the subjective sense of identity confusion. The relationship between internally-guided decision making and the subjective sense of identity has not been explored, and this point needs to be examined in future studies. Meanwhile, the salience of identity confusion is in line with the findings of the previous studies on identity. Indeed, identity confusion was related to more diverse dimensions of psychological well-being and adjustment than identity synthesis^32,48^. These findings suggest that the *lack of temporal coherence* in one’s sense of identity may be especially problematic^32^. Taken together, our results provide a novel insight into the relationship between alpha LRTC and identity; thus, mechanisms for the link between LRTC and identity might be different between identity synthesis and identity confusion.

In addition to our focus on the alpha range, in the analysis for descriptive purposes, we observed that the frontal theta LRTC correlated with identity synthesis, and the centroparietal beta LRTC correlated with identity confusion (Fig. 3). The directions of the correlations were consistent with our hypotheses, but the frequency ranges and channels were outside the scope of prediction. Although the details of the underlying mechanism of these correlations are unclear, our results suggest that identity synthesis and confusion are associated with different spatiotemporal neural substrates, at least for the sample used by us.

Frontal theta relates to the activity within the medial prefrontal cortex^56,57^, which is a part of the default mode network (DMN). The DMN is the first identified resting-state network^58,59^, and has an essential role in the internal/self-oriented cognitive process^26,37,60,61^. Given that the LRTC emerges from the network with balanced excitatory and inhibitory connections^11,12^, it is possible that the well-organized theta range activity within the medial prefrontal cortex relates to the subjective sense of identity synthesis.

Regarding the beta ranges, Nikulin et al.^62^ reported that schizophrenia showed attenuation of LRTC, including the occipitoparietal channels. Schizophrenia is thought to be characterized by increased neuronal noise based on a reduction in excitatory and inhibitory synaptic connections^63^. Given that schizophrenia is associated with identity confusion^64,65^, we postulate that identity confusion might be associated with reduced excitatory and inhibitory connections, similar to that observed in schizophrenia.

### Limitations and future implications

Although we revealed the link between neuronal temporal consistency and subjective sense of identity for the first time, the following limitations should be considered. First, the sample of the present study was limited to undergraduate and graduate students in Japan, which may inhibit the generalizability of our findings. LRTC is known to change with age^8^, and careful consideration of cultural differences is essential for the study of identity^32,66^. Considering the correlation between LRTC and identity confusion was not strong (*rho* = − 0.27), it is essential to confirm reproducibility using a larger sample population in further studies.

Second, the largest time scale to calculate DFAe was 16 s, similar to that performed by Meisel et al.^13^; thus, the LRTC with a larger time scale was not reflected in the present results. We applied an automatized preprocessing procedure as outlined previously^20^, in order to avoid the effect of artifacts. In cases using epochs with a longer time (e.g., 30 s), the proportion of the rejected epochs are increased, which results in a decreased signal–noise ratio. We selected 20 s epochs for the present dataset in order to keep the available data after applying preprocessing. Additional studies are needed to examine the relationship between LRTC with longer time scales and identity.

Third, the tripartite relationship between LRTC, internally-guided decision making, and identity remains unclear. While Nakao et al.^20^ reported the most obvious correlation between LRTC and internally guided decision making in the alpha band, the present data showed that LRTC in the theta and beta bands had close relationships with identity (see Figs. 2 and 3). Those findings suggest that the relationships between LRTC and internally-guided decision making or identity do not necessarily capture an analogous brain-mind association. These tripartite relationships remain an issue for future consideration.

Fourth, the correlation in our main finding was not strong (*rho* = − 0.27; Fig. 2(b)). This implies that the relationship between LRTC and identity is neither direct nor straightforward. As identity is a complex construct, only one measure (i.e., the EPSI in this study) may not provide a comprehensive picture of its association with neural noise. The absence of EPSI compatible measures for sense of consistent self across time also limited our study. In future research, it would be helpful to use measures developed from another perspective on identity. Specifically, there is a set of measures for one’s work of maintaining and revising the sense of identity, that is, exploring options and making commitments regarding important life choices (e.g., academic goals, future careers, and relationships). Concretely, the EPSI is not the only scale that assesses a subjective sense of identity; others such as the Dimensions of Identity Development Scale^67^, which tap into exploration and commitment, must also be included in future studies.

## Conclusion

In the current study, we demonstrate that the neuronal noise of intrinsic activity, as indexed by its frontocentral alpha LRTC, is related to the subjective sense of identity confusion. At the same time, although reported for descriptive purposes only, negative correlations between LRTCs and identity confusion were also found in the occipitoparietal beta band, while positive correlations between LRTC and identity synthesis were found in the frontal theta band. Thus, less noise and more temporal consistency in the intrinsic activity, as indexed by a higher LRTC in different bands and channels, may be involved in lower degrees of random noise at the psychological level of sense of identity (i.e., less confused and well-synthesized identity). Our data provide further evidence that the LRTC of intrinsic activity might serve as a noise suppression mechanism at the psychological level.

## Methods

### Participants

Based on an a prior power test for one-tailed correlation (statistical power = 0.80, type I error probability = 0.05, effect size = 0.30) and the predicted sample reduction in EEG preprocessing, 87 healthy undergraduate and graduate students (47 male; age range, 18–36 years; mean age, 21.5 years) were recruited from Hiroshima University. We confirmed the following based on the participants’ self-reports: All participants were native Japanese speakers, right-handed, with normal or corrected-to-normal vision. All were free of neurological and psychiatric disorders, and none of the participants were medicated, habitual drinkers, or smokers. We instructed all participants to avoid consuming caffeine, alcohol, or nicotine within the 3 h before starting the experiment, which they followed. All experimental protocols were conducted in accordance with the relevant guidelines and regulations approved by the Ethical Committee of the Graduate School of Education, Hiroshima University. Written informed consent was obtained from each participant prior to the experiment, and each participant was paid a small fee for participation.

After the automatized preprocessing of resting-state EEG (see below for more details), 16 participants, who remained less than 10 epochs (20 s epochs with 75% overlapped) data, one participant who reported that he slept during the resting-state EEG recording, and two participants with missing questionnaire data were excluded. Thus, a total of 68 participants (34 male; age range, 18–36 years; mean age = 20.96 years) were included in the final analyses.

The data used in the present study were collected as a part of an ongoing project at the Cognitive Psychology Laboratory of Hiroshima University to investigate associations among resting-state EEG, behavioral performance, and personality^68^.

### Procedures

Prior to conducting the experiment, participants were instructed about the experimental procedure, read and signed the consent form. Following EEG electrode placement, participants were seated on a comfortable chair in a quiet shielded room. Participants used a chin rest to maintain their head position and minimize the movement during recording.

Participants performed 5 min of eyes-closed resting-state periods, in which participants were instructed to relax. After the recording, participants completed a short questionnaire, which involved items relating to whether they kept closing their eyes, whether they remained wakeful during the recording, and the nine-point scale subjective-rating of arousal level during the resting-state recording in line with previous studies^20,69^.

Identity synthesis and confusion were assessed by the identity subscale of the Erikson Psychosocial Stage Inventory^46^ (Japanese version validated by Hatano et al.^47^). The scale consists of 12 items (6 items for identity synthesis and 6 items for identity confusion), and the response scale ranges from 1 (strongly disagree) to 5 (strongly agree). The sample items include the following: For synthesis, “I know what kind of person I am” and “I've got a clear idea of what I want to be”; and for confusion “I feel mixed up” and “I can't decide what I want to do with my life”.

The degree of depressive tendency was measured using the Center for Epidemiological Studies Depression Scale (CES-D^50^; Japanese version validated by Shima et al.^51^) in order to confirm the external validity of identity synthesis and confusion. The scale consisted of 20 items, and the sample item included ” I was bothered by things that usually don’t bother me”. Participants rated how often they had felt this way during the prior week by using the 4-point response scale that ranged from “rarely or none of the time” to “most or all the time”.

### EEG recording

EEGs were recorded using 63 silver-silver chloride cup active wet electrodes (actiCAP; Brain Products GmbH., Gilching, Germany), placed at AF3, AF4, AF7, AF8, C1, C2, C3, C4, C5, C6, CP1, CP2, CP3, CP4, CP5, CP6, CPz, Cz, F1, F2, F3, F4, F5, F6, F7, F8, FC1, FC2, FC3, FC4, FC5, FC6, FCz, Fp1, Fp2, FT10, FT7, FT8, FT9, Fz, O1, O2, Oz, P1, P2, P3, P4, P5, P6, P7, P8, PO10, PO3, PO4, PO7, PO8, PO9, POz, Pz, T7, T8, TP7, and TP8 according to the extended International 10–10 Systems. A ground electrode was placed at AFz. Although the reference electrode was positioned on the tip of the nose during on-line recording, all electrodes were later re-referenced to the average reference. Eye movements and blinking were monitored with electrodes above and below the right eye (vertical electrooculogram; VEOG). The electrode impedance, measured after the placement of electrodes, was less than 20 kΩ, which was in accordance with the recommendation of Brain Products^70^. The EEG signals were amplified with a low-pass of 250 Hz, and were digitized at a sampling rate of 1000 Hz using a Brain Amp DC-EEG recorder (BP-01110; Brain Products GmbH., Gilching, Germany).

### EEG preprocessing

Preprocessing of resting-state EEG data was conducted by EEGLAB toolbox (ver.14.1.2b)^55^ running on Matlab 9.4.0 (The Mathworks Inc.). The EOG channels were removed from the data, and the automatized artifact rejection methods were based on those used in a previous study^20^. Data were down-sampled to 250 Hz and were filtered using a finite impulse response (FIR) filter, with a low-pass of 50 Hz and a high-pass of 1 Hz. The ‘clean_rawdata’ EEGLAB plug-in was used to remove bad channels and non-stationary artifacts with the Artifact Subspace Reconstruction method (ASR)^71^. Channels with a flat line longer than 5 s, with less than 0.85 correlation with their reconstruction from neighboring channels, or those with line noise more than 4 standard deviations (*SD*) from the other channels, were removed. The mean number of the removed channels across participants was 2.03 (*SD* = 1.91). A threshold of 20 *SD* was used for non-stationary artifact correction. The time windows were rejected if the artifact contaminated more than 25% of the channels, even after applying ASR. The rejected channels were spherically interpolated using the ‘pop_interp’ function. EEG data were re-referenced to an average reference.

Independent component analysis (ICA) was used for the rejection of stationary artifacts (e.g., eye movement) from the EEG data. An adaptive Mixture of ICA (AMICA)^72^ was conducted once for each participant’s data. The number of ICs was determined by the rank of the EEG data matrix. An equivalent current dipole was estimated for each IC (DIPFIT EEGLAB plug-in using the Fieldtrip toolbox). The selection of ICs and fitting of the bilateral dipole were performed using the ‘fitTwoDipole’ EEGLAB plug-in^73^. The ICs were rejected based on the criteria of dipoles with more than 15% residual variance^74^, and the outside brain was rejected. On average, 41.78 (*SD* = 5.17) ICs were rejected from the data of each participant, and 19.19 ICs (*SD* = 4.99) were retained. The retained number of ICs was similar to that of the previous study^20^. The remaining ICs were back-projected onto the scalp electrodes to obtain artifact-free EEG data. To leave as many epochs as possible, and thus to increase the signal–noise ratio, the EEG data were segmented into 20-s epochs as outlined previously^13^. The epochs were 75% overlapped, and if the epochs contained a boundary as the result of ASR time window rejection, the epochs were rejected. The data of 16 participants, which retained less than 10 epochs after the artifact rejection, were removed from the following analyses. The averaged retained number of epochs for the 68 participants was 38.29 (*SD* = 13.84).

### LRTC and measurement confirmation

Detrended fluctuation analysis (DFA)^1,2,44,45^ was applied to quantify long-range temporal correlations (LRTC) using the Neurophysiological Biomarker Toolbox (NBT ver. 0.5.5)^2^. A larger DFA exponent (DFAe), within 0.5 to 1.0, reflects a high degree of long-range temporal correlation. A 62-degree FIR filter was applied to extract the alpha range (lower cut-off frequency = 8 Hz, upper cut-off frequency = 13 Hz) EEG time series for each artifact-free 20-s epoch. The Hilbert transform was used to calculate the amplitude envelope, and the cumulative sum of the envelope was computed. Twenty-six time windows of log-linearly increasing length from 1 to 20-s were extracted with a 0% overlap from each epoch, and each of these time window was linearly detrended using a least-squares fit; this was set to 0% overlap, since the epoch itself was extracted with the 75% overlap to increase the number of remaining epochs during preprocessing. The *SD* of the detrended data was computed for each time window. The average of the *SD* across all the time windows of the same size from all epochs were used as the fluctuation function, *F*(t). *F*(t) was plotted for all time window sizes on the logarithmic axes. The exponent of DFA (i.e., the linear slope of the trend line in the 2–16 s range; DFAe) was estimated according to Meisel et al. (see Fig. 1)^13^. The degree of overlap (i.e., 75%) was in line with that of previous studies^18,20^, and the overlap increased the accuracy of the estimate of *F*(t)^2^.

To examine the reliability of the DFAe, the same numbers of the first and second halves of the available non-overlapping 20-s epochs were extracted from each participant and averaged. The intraclass correlation coefficient (ICC; one-way random effects, absolute agreement, and multiple measurements)^75^ values were calculated.

Furthermore, surrogate data analyses were conducted to examine whether the observed DFAe results reflected the temporal structure of the phase. Although the surrogated data have an identical amplitude distribution to the original time series, they do not have the same temporal correlation as the original data. Thus, the amplitude adjusted Fourier transform (AAFT) algorithm^76^ was applied using the Chaotic Systems Toolbox in order to surrogate the data^77^. In this algorithm, random data derived from a normal distribution was sorted by the rank of amplitudes of the original time series data. The phase of the data was randomized, and then the original data were sorted by the rank of the amplitude of the phase randomized data. Surrogating was conducted 2000 times for the data of each participant in line with our previous study^20^, which constituted of a larger number of iterations than that done in other studies^16,78^. By following the methods described by Nakao et al. (2019), two types of mean surrogated data were calculated for the DFAe. The first was the mean surrogated DFAe, which was calculated by averaging the DFAe across participants for each of the 2000 surrogated data. The mean surrogated DFAe was then used to calculate a critical value, which corresponds to a higher percentile of the mean surrogated DFAe^76^. The second type was the mean surrogated DFAe for each participant, which was calculated by averaging the DFAe across the 2000 surrogated data for each participant. These data were used to examine the relationship with the EPSI scores.

### Correlation analyses

Spearman’s partial rank correlation coefficient (*rho*) was used to avoid the effect of outliers. Age, gender, body mass index (BMI), self-reported arousal level during resting-state EEG recordings, and the retained number of 20-s epochs were entered as the control variables in the partial correlation analyses since those are known to be potential confounding factors of resting-state data^79^. Identity synthesis was also entered as the control variable in the correlation analysis for identity confusion, and vice versa^32^. The BMI was calculated from the self-reported height and weight. The retained numbers of 20-s epochs were included as the control variables, since this can affect the signal to noise ratio of EEG data.

Since we had no specific hypothesis about which of the identity factors (e.g., synthesis and/or confusion) would correlate with frontocentral alpha DFAe, Bonferroni correction was applied for the two correlation analyses between identity factors and DFAe to avoid type I error inflation.

The DFAe for theta (4–8 Hz), beta (13–30 Hz), and gamma (30–45 Hz) bands were also calculated, and the correlation results were reported for descriptive purposes.

## Data Availability

The data of each participant (age, gender, BMI, arousal level, the available number of epochs, EPSI scores, CES-D score, and LRTC in theta, alpha, beta, and gamma at each electrode) are available from https://mfr.osf.io/render?url=https://osf.io/eawfr/?direct%26mode=render%26action=download%26mode=render.
